# The Matrix Effect in the RT-PCR Detection of SARS-CoV-2 Using Saliva without RNA Extraction

**DOI:** 10.3390/diagnostics12071547

**Published:** 2022-06-25

**Authors:** Orlando Morais, Manuel Rui Alves, Carla Ramos, Fernando Ferreira, Paulo Fernandes

**Affiliations:** 1 Escola Superior de Tecnologia e Gestão, Instituto Politécnico de Viana do Castelo, Rua Escola Industrial e Comercial de Nun’Álvares, 4900-347 Viana do Castelo, Portugal; omorais@estg.ipvc.pt (O.M.); mruialves@estg.ipvc.pt (M.R.A.); cramos@estg.ipvc.pt (C.R.); 2 CISAS, Escola Superior de Tecnologia e Gestão, Instituto Politécnico de Viana do Castelo, Rua Escola Industrial e Comercial de Nun’Álvares, 4900-347 Viana do Castelo, Portugal; 3 ACES Cávado III Barcelos/Esposende, Rua Dr. Abel Varzim, 4750-253 Barcelos, Portugal; facferreira52@gmail.com

**Keywords:** SARS-CoV-2, saliva, RT-PCR, Ct values, sensitivity, detection, COVID-19, inhibition

## Abstract

The present work focuses on the detection of SARS-CoV-2 in saliva, contributing to understanding the inhibition effect of the matrix and its influence on the results. Detection of viral genes ORF1ab, N, and E was performed by RT-PCR using saliva directly in the reaction without RNA extraction. Different amounts of saliva were spiked with increasing amounts of viral RNA from COVID-19 patients and subjected to RT-PCR detection. In parallel, 64 saliva samples from confirmed COVID-19 patients were used in two different amounts directly in the RT-PCR reaction and their results compared. The presence of saliva in the RT-PCR always causes a positive shift of the Ct values, but a very high between-person variability of its magnitude was obtained, with increases ranging from 0.93 to 11.36. Viral targets are also affected differently depending on the initial number of viral particles. Due to inhibitors present in saliva, the duplication of sample volume causes only 48 to 61% of the expected Ct value decrease depending on the viral target gene. The use of saliva has advantages, but also limitations, due to potential inhibitors present in the matrix. However, the choice of the target and the right amount of sample may significantly influence the results.

## 1. Introduction

Saliva, due to its convenience, low cost, and easy collection, has been considered an interesting specimen for use in the diagnostics of respiratory viral infections [1,2,3], including SARS-CoV-2 [1,4,5,6,7,8,9,10,11,12,13]. However, documented results use different systems and methods, making the use of this specimen difficult to assess in terms of effectiveness and reliability. Additionally, as recently reviewed, some contradictory findings have been described [14]. Some authors use saliva to extract nucleic acids that are used in subsequent PCR reactions [2,12,15], while others demonstrated that it is possible to use saliva directly in the PCR reaction to detect viral RNA, in a much faster and less costly process [16,17,18]. However, inhibitors of PCR reactions can be found in many different matrixes, such as saliva, which may affect the polymerase activity as well as other components of the system, interfering directly with target nucleic acids or by quenching fluorescence [19,20]. Saliva is not an exception and the presence of inhibitors in this specimen has been reported already for a long time [21].

In the present work, the effect of saliva used directly without extraction, on the RT-PCR detection of SARS-CoV-2 genes E, N and ORF1ab is assessed, namely its influence on the Ct values, which is a determinant for the sensitivity of the method.

## 2. Materials and Methods

### 2.1. Sample Collection

COVID-19 positive saliva samples were obtained from patients oriented to perform the detection of SARS-CoV-2 and they were previously informed in writing about the purpose and procedure of the study and consented to participate through the act of providing the samples. This work was considered exempt from review by an institutional ethical review board, because it comprises the use of completely anonymized specimens obtained voluntarily and informed.

The saliva samples were collected under the supervision of a healthcare worker from ACES Cávado III Barcelos/Esposende, by just letting the mouth drop accumulated saliva into empty sterile sputum containers. All samples were stored at 4 °C until processed. 

### 2.2. Preparation of Saliva Samples Prior to RT-PCR

Saliva samples were prepared to be used directly in the RT-PCR reaction using a 20 mg/mL proteinase K solution (NZYTech, Lisbon, Portugal) and heating at 95 °C for 5 min [16].

### 2.3. Real-Time Reverse Transcriptase-Polymerase Chain Reaction Assay (RT-PCR) for SARS-CoV-2

Samples were tested for SARS-CoV-2 using a Novel Coronavirus (2019-nCoV) RT-PCR Detection Kit (Shanghai Fosun Long March Medical Science, Shanghai, China) and the CFX96 real-time PCR system (BioRad, Hercules, CA, USA) in accordance with manufacturer’s instructions. 10 μL of saliva samples, undiluted and 1:1 diluted with RNase free water (PanReac AppliChem, Darmstadt, Germany), prepared as previously described, were added into 20 μL of the reaction mixture. Reactions were incubated at 50 °C for 15 min and 95 °C for 3 min followed by five cycles at 95 °C for 5 s and 60 °C for 40 s and 40 cycles at 95 °C for 5 s and 60 °C for 40 s, targeting SARS-CoV-2 genes N, E, and ORF1ab. At the end of each of the last 40 cycles, the signals of FAM, JOE, ROX, and Cy5 fluorescence signals were registered. The internal control present in all the reactions was detected using the Cy5 fluorescence signal. A cycle threshold value (Ct value) less than or equal to 36 was defined as a positive test result, and more than 36 or no value was considered to be negative.

### 2.4. Real-Time Reverse Transcriptase-Polymerase Chain Reaction Assay (RT-PCR) in the Presence of Different Amounts of Saliva

A pool of RNA extracted from nasopharyngeal swabs from confirmed COVID-19 patients was serial diluted (1:10) with RNase free water. A set of triplicate RT-PCR reactions were performed, as described before, in the absence of saliva and in the presence of 2, 4 and 6 µL of saliva (previously tested as negative for the presence of SARS-CoV-2) and pre-treated as previously described. 

### 2.5. Statistical Analysis

Data were analyzed following conventional methodologies [22], mainly as available in the R base package [23]. Binomial tests were used to compare proportions. Parametric and non-parametric analysis of variance, respectively ANOVA with post-hoc Tukey HSD tests, and Kruskal–Wallis test followed by post-hoc Wilcoxon rank-sum tests, were used for comparisons between groups. Anderson–Darling tests (nortest package) were used to assess data normality. All graphs but one (Figure 2) were produced using routines written by the authors using the R language. Excel was used as a general data organization and analysis tool. RT-PCR efficiencies were determined using the on-line tool Real-Time PCR Miner (version 4.0), according to a previously described and published method [24].

## 3. Results

### 3.1. Detection of SARS-CoV-2 in Saliva Spiked with Viral RNA from COVID-19 Patients

**Figure 1:**
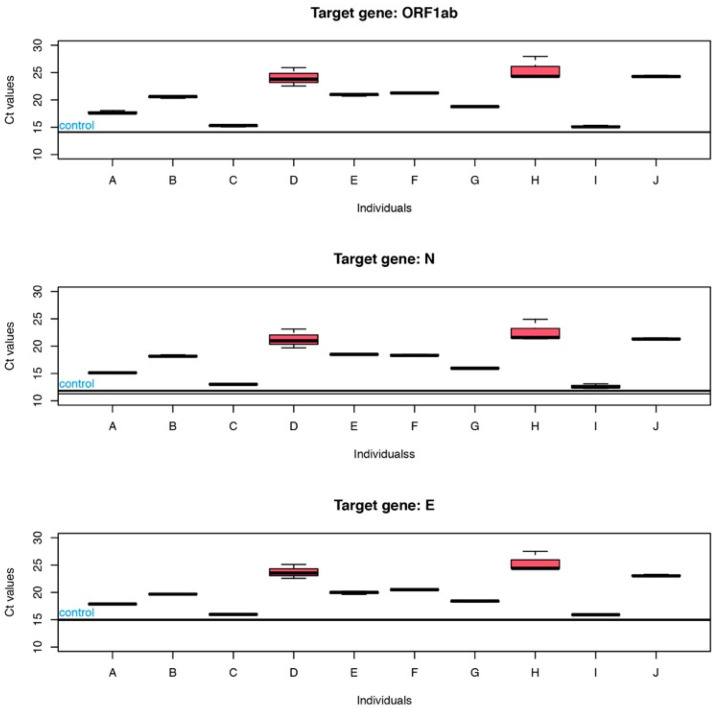
Ct values relative to ORF1ab, N and E genes, for reactions in the presence of saliva (5 µL) from ten different individuals (A to J). Horizontal lines, identified as control, refer to the Ct values of control reaction with no saliva.

Ten saliva samples from different individuals confirmed as COVID-19 negative were spiked with the same amount of RNA from COVID-19 patients. After RT-PCR, the Ct values of each individual reaction were compared to the Ct of the reaction without saliva. A positive shift of Ct values was observed for all samples when saliva was present, as shown in Figure 1. 

The mean positive shift observed in the presence of saliva was 6.23 ± 3.65 (median of 6.62), 6.04 ± 3.47 (median of 6.62) and 5.07 ± 3.22 (median of 4.85), respectively, for ORF1ab, N, and E genes. A high between-person variability was obtained with a positive shift in Ct values ranging from 0.93 for individual “I” to 11.36 for individual “H”. These results were confirmed by a one-way ANOVA with individuals as the main effect, showing that there are highly significative differences (*p* ≈ 7.2 × 10^−12^) in the increase in Ct depending on individual’s saliva. Kruskal-Wallis tests provided similar conclusions.

**Figure 2:**
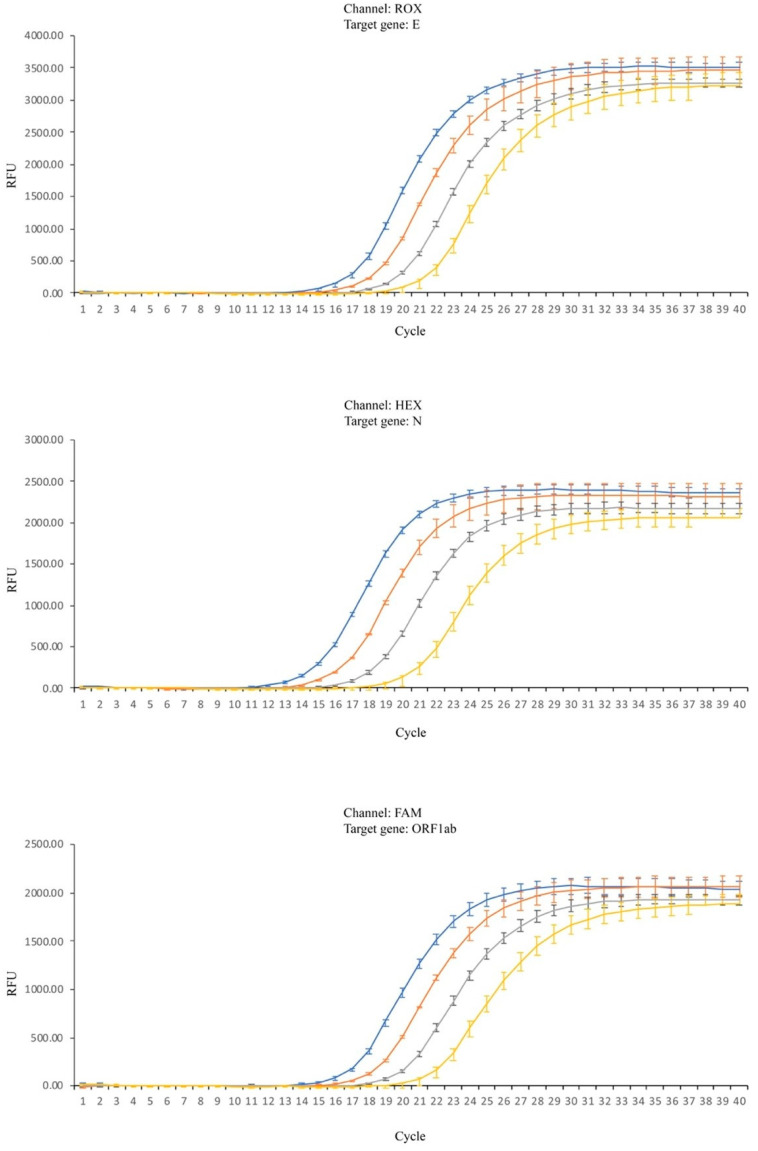
RT-PCR amplification reactions for the three viral target genes. Blue lines—no saliva; orange lines—2 µL of saliva; gray lines—4 µL of saliva; yellow lines—6 µL of saliva; Standard deviation (SD) represented by vertical bars.

Despite these results, further studies were performed in order to perceive the magnitude of the influence of the matrix in the RT-PCR. Reactions were performed using the same amount of viral RNA undiluted (referred to as 1:0) and in three different dilutions (1:10, 1:100 and 1:1000) combined with different amounts of a saliva sample displaying a Ct shift within the median value previously determined. In Figure 2, it is clearly seen that increasing the amount of saliva causes a rightward shift of all curves and this effect is particularly visible when the targets are the ORF1ab or N genes.

The presence of saliva influences the Ct of the reaction, but also the reproducibility of the essay, as the amplitude of the results tends to increase when high volumes (6 µL) of saliva are used. 

**Figure 3:**
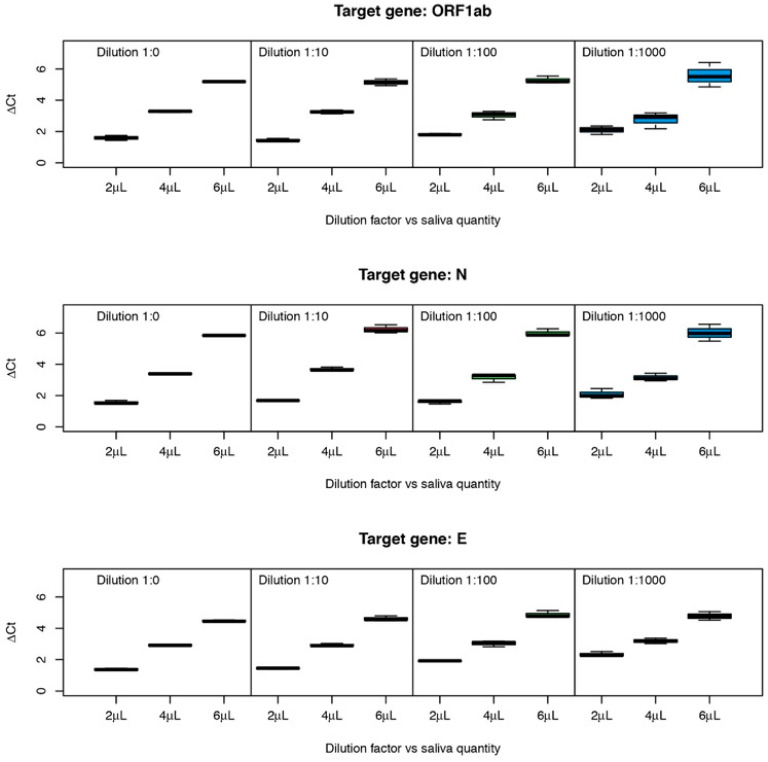
ΔCt values obtained for RT-PCR reactions using different amounts of saliva and initial viral RNA concentrations.

Figure 3 represents mean ΔCt values (in relation to the reaction without saliva) for genes ORF1ab, N, and E, with two variation factors: saliva quantity (2, 4, and 6 µL) and four dilutions (1:0, 1:10, 1:100 and 1:1000). There is an evident increase in ΔCt values dependent on the increase in saliva quantity. 

To further investigate the effect of the two factors and possible interactions, ΔCt values were analyzed by a two-way ANOVA for each target gene, with saliva quantity and dilution as main factors and with interaction effects. ANOVA *p*-values are reported in Table 1. 

There is an obvious effect on ΔCt values due to increasing amounts of saliva (*p* < 2 × 10^−16^ for all genes), as clearly suggested by the observation of Figure 2 and Figure 3. Concerning the effect of the amount of initial RNA, the dilutions 1:0, 1:10, 1:100, and 1:1000 for gene ORF1ab have no significant effect. For gene N, the dilution factor is also not significant although it has a very low *p*-value (*p* = 0.0599). For these two genes, the interaction between amount of saliva and dilution (amount of initial RNA) is very close to significance (0.0648 and 0.0552, respectively). For gene, E the dilution factor and the interaction are very significant. Tukey post-hoc tests showed that dilutions 0–10 and 100–1000 are not significantly different, while dilutions 10–100 are significantly different (*p* = 0.004), showing that for this gene, the inhibition power of saliva is stronger when the concentration of RNA in the sample is lower.

### 3.2. Saliva Influence on RT-PCR Efficiency

To assess the inhibitory effect of saliva in the amplification reactions, PCR efficiencies were calculated for each individual reaction using a published algorithm [24], which, after fitting the RT-PCR raw data, uses a four-parameter logistic model to identify the exponential phase of the reaction.

Mean efficiencies were different for the three viral targets, with 0.945 ± 0.044 for the ORF1ab gene, 0.944 ± 0.014 for the E gene, and 0.893 ± 0.013 for the N gene. The presence of saliva does not seem to influence the efficiency of the reactions since, in the presence and absence of saliva, the efficiencies of each reaction generally remain within the levels considered normal for an RT-PCR reaction.

### 3.3. Detection of SARS-CoV-2 Directly from the Saliva of COVID-19 Patients

Saliva samples were analyzed directly in RT-PCR reactions using 10 µL of COVID-19 confirmed patients’ saliva (positive nasopharyngeal swabs—NPS), undiluted and 1:1 diluted with RNase free water (equivalent to use just 5 µL in the RT-PCR reaction instead of 10 µL). Four positive samples were detected only when using the undiluted sample. The comparison of the results (detected/not detected) was carried out through a binomial test considering the number of successes as the number of different conclusions when using diluted and undiluted saliva. The null hypothesis was “H_0_: there is no difference between the results”.

When using only 5 µL of saliva (diluted sample), the number of successes decreased from 52 to 49, which corresponds to a decrease of 5.7% in sensitivity for values between 0.643 and 0.862, with the value of 0.766 as the most likely (*p* = 2.436 × 10^−5^). It is worth noting that the wide confidence interval for this proportion is due to the use of the binomial distribution to test proportions, which always happens with samples relatively small, typical of these kinds of studies. There was a situation where the positive was detected only when the diluted saliva was used. Wilcoxon signed-rank tests were used to compare the Ct values for each gene of NPS samples with the values of the RT-PCR performed with diluted and undiluted saliva samples, with the hypothesis “H_0_: there is no difference”. The results obtained show that the differences are highly significant for genes ORF1ab (*p* = 6.291 × 10^−5^) and N (*p* = 0.00329), while for gene E differences are only close to significance (*p* = 0.0672). In view of these results, a study of the observed differences was carried out. Differences in Ct values (diluted vs. undiluted samples) were calculated and tested for normality using a Anderson–Darling test, which showed that there is no reason to think that the observed differences follow a normal distribution (*p* = 0.00152 for gene ORF1ab, *p*= 2.639 × 10^−6^ for gene N and *p*= 0.00536 for gene E), indicating that the magnitude of difference is not random.

The results show an increase in Ct values of the three viral genes, with a median of 0.30 for gene N (IQR 1.00), 0.20 for gene E (IQR 0.99) and 0.49 for gene ORF1ab (IQR 0.96). It also becomes clear that differences increase with increasing Ct values.

## 4. Discussion

Recent works using saliva as a specimen to detect SARS-CoV-2 demonstrates that it is a matrix that offers some performance limitations. When its results are compared with the ones obtained using NPS samples, a good detection concordance between the two types of samples is obtained [14]. However, a significative increase in the Ct values can be observed, although its magnitude is variable as observed by different authors [16,25,26]. A previous work on the comparison of dual samples (saliva and NPS) from the same patient has shown an average increase of 7.3 in Ct values [27]. This figure must be observed with caution since it cannot be assumed that for any specific patient the viral loads obtained from NPS and saliva samples are equivalent, as many factors can influence results, such as efficacy of sampling [28], stage of infection [26], and shedding of virus in different areas of the body. However, it can always be argued that this significative increase in Ct values may result in a lower performance of the detection reaction.

Due to the observed increase in Ct values and the already known saliva inhibitor potential, the present work attempted to observe and quantify the effect of saliva in the detection performance of three viral targets, namely ORF1ab, E, and N genes. It was seen that the amount of saliva may, in fact, be relevant due to the presence of inhibitors, and therefore the increase of its volume in the reaction may have an opposite effect in terms of viral detection despite the increase in the amount of target template.

Although the presence of inhibitor substances may have an impact on the RT-PCR efficiency, in this work, no significative differences were found in the presence or absence of saliva. In fact, some inhibitor molecules such as IgG, which interfere with primer annealing, may cause an increase in the Ct values without causing significative changes to the reaction’s efficiency [20,29,30]. Furthermore, if the inhibitor molecule is thermolabile its inhibitor potential decreases as the number of PCR cycles increases, and after a certain point, the reaction may proceed as in the absence of inhibitor, without affecting the log-linear phase of the amplification but delaying its occurrence and causing a positive shift in the Ct value of the reaction.

As could be observed in Figure 2, the amplification curves are clearly displaced to the right in the presence of saliva and a positive shift in the Ct values is clear and consistent with the increase in the amount of saliva in the reaction. Although a shift of the Ct values was observed for all the viral genes tested, its magnitude is variable and target dependent. There also seems to be some influence of saliva on reproducibility, as the samples that present larger Ct shifts are the ones presenting larger variance of results (as can be observed in Figure 1, individuals D and H, and amplification curves in the presence of 6 µL of saliva, Figure 2). For genes N and ORF1ab, the amount of initial RNA does not seem to influence the inhibitory power of saliva as suggested by the two-way ANOVA analysis. However, when gene E is the target, the amount of initial RNA influences the magnitude of the shift, increasing with the decrease in the initial concentration. An increase in 3.32 Ct values corresponds to a decrease of about 10 times the amount of the initial template. As the observed increase is over 6 Ct values, this corresponds, roughly, to say that the presence of as much as 6 µL of saliva in the reaction delays the exponential amplification as if there were 100 times less quantity of initial template. This may have a significant impact on the detection sensitivity as, for samples with lower viral load, the increase in the Ct values caused by the presence of inhibitors in saliva may throw the Ct to values above the threshold used for considering a sample as positive. As previously shown (Figure 1), this effect is highly variable and person dependent, and therefore an estimation of the impact of the matrix on the RT-PCR results, as presented, should only be viewed as a rough estimation due to the variability of the effect between different individuals.

Saliva composition can be influenced by many factors, such as individual hydration, medication, lighting, smoking, gender, and circadian cycle [31], which may justify the between-person variability observed in the present work. These factors affect saliva’s inhibitory effect and are difficult to control, challenging the use of this kind of matrix. However, some limitations of the present study must be acknowledged, mainly the fact that only ten saliva samples of healthy individuals were used to assess saliva’s inhibitory power. Despite the low number, the results clearly demonstrate not only the variability of the magnitude of the effect but also its consistency among the tested samples. However, although within-person variability was not tested, due to the factors affecting saliva composition, it is highly probable that some variation might also occur.

The use of undiluted instead of diluted saliva of COVID-19 patients results in a shift of the Ct values which is significantly less than the 1 Ct value theoretically expected, due to the duplication in the amount of viral mRNA in the undiluted sample. The difference to the theoretical value is explained by the presence of inhibitory substances, which increases with the amount of volume of saliva used. However, the results obtained clearly show that although an inhibition occurs in the presence of saliva, the dilution of the sample will not solve this problem, as the increase in the amount of sample seems to compensate for some of the matrix’s inhibitory potential. In fact, a lower sensitivity was obtained with the decrease in the amount of sample. The effect of the amount of saliva on the shift of Ct values seems to be influenced by the viral load, as suggested by graphs in Figure 2, showing a trend in the increase of the difference in Ct values accompanying the decrease in sample’s viral load. The target most affected seems to be ORF1ab, the most conserved but also the least sensitive of all the three viral targets tested [32], presenting a median increase in Ct value of 0.49 which is about half of the expected value.

The potential of saliva in molecular diagnosis using PCR has been studied for several years, but the inhibition that this matrix exerts in the reaction, is not negligible and should be carefully considered. Some studies point out that subjecting saliva to high temperatures can contribute to a decrease in its inhibitory power of PCR reactions [21,33]. The choice of the viral targets to be used in the detection reaction is also relevant, as ORF1ab, E, and N genes present different behaviors in the presence of the saliva inhibitors.

## Figures and Tables

**Table 1:** *p*-values for two-way ANOVA for three target genes with two factors (saliva quantity and dilution factor) and factor interactions.

Gene	Factor		Factor Interaction
	Saliva Quantity	Dilution	
ORF1ab	<2 × 10^−16^	0.6012	0.0648
N	<2 × 10^−16^	0.0599	0.0552
E	<2 × 10^−16^	3.56 × 10^−7^	0.0017

## Data Availability

Not applicable.
